# Experimental Study on the Enhanced Oil Recovery Mechanism of an Ordinary Heavy Oil Field by Polymer Flooding

**DOI:** 10.1021/acsomega.2c08084

**Published:** 2023-04-04

**Authors:** Fengjiao Wang, He Xu, Yikun Liu, Yingnan Jiang, Chenyu Wu

**Affiliations:** † Laboratory of Enhanced Oil Recovery of Education Ministry, Northeast Petroleum University, Daqing, Heilongjiang 163318, China; ‡ Luliang Operation Area of Petro China Xinjiang Oilfield Company, Karamay, Xinjiang 834000, China; § Daqing Oilfield Production Engineering Research Institute, Daqing, Heilongjiang 163453, China; ∥ Heilongjiang Provincial Key Laboratory of Oil and Gas Reservoir Stimulation, Daqing, Heilongjiang 163453, China

## Abstract

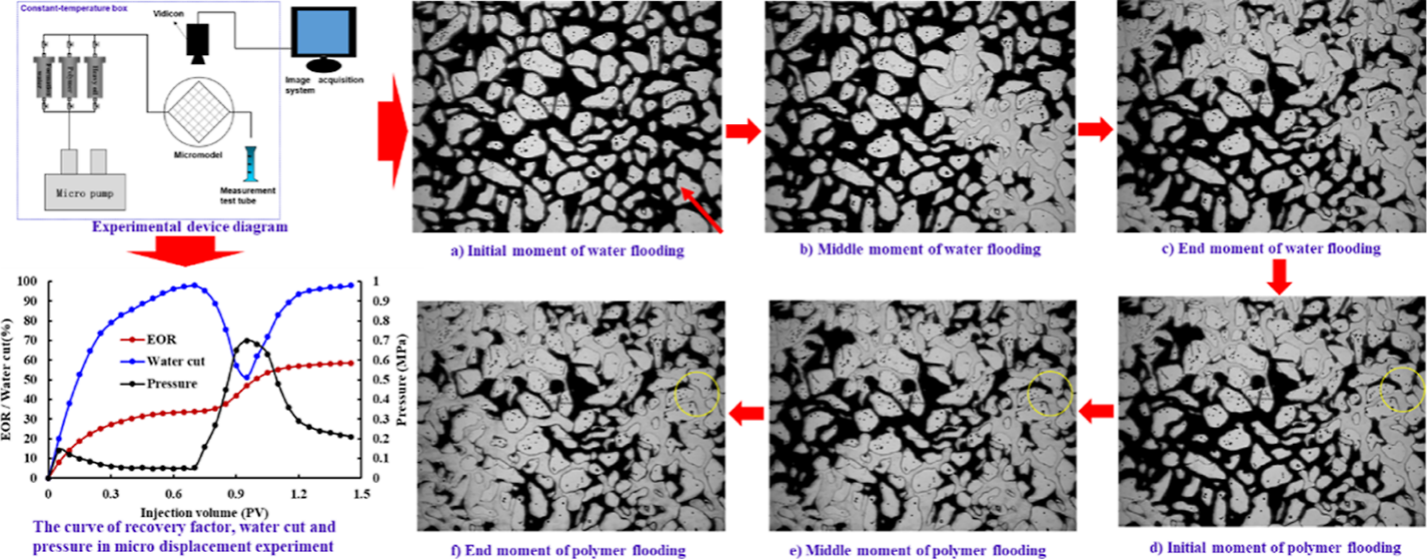

It is widely known that in the water flooding development process of ordinary heavy oil, the fingering phenomenon is obvious, there are a lot of unswept areas, and absolutely, the recovery is really very low. In addition, for some shallow and thin ordinary heavy oil reservoirs limited by the geological conditions of the reservoir, the thermal recovery technology also has serious heat loss and high development cost. Therefore, there is an urgent need to transform the development and further improve the enhanced oil recovery (EOR). In this paper, the mechanism of EOR by polymer flooding was investigated for high-porosity and high-permeability terrestrial ordinary heavy oil reservoirs. Through laboratory experiments, we analyzed the characteristics of oil–water relative permeability curves, mobility control ability, and microscopic seepage characteristics during polymer flooding of ordinary heavy oil reservoirs. On this basis, the effect of the mobility ratio on seepage characteristics and the mechanism of EOR enhancement were clarified. The results show that the polymer can effectively improve the mobility control effect of the displacing fluid. As the polymer solution and ordinary heavy oil have the characteristics of high viscosity and low mobility, there is a minimum mobility ratio in the process of polymer flooding. Namely, the characteristics of dual low mobility exist in the process of polymer flooding for the ordinary heavy oil. It effectively enhances the profile control and plugging ability of the polymer, thus expanding the sweep volume of larger pores and improving the displacement efficiency of smaller pores. Based on the two factors mentioned above, it is found that the dual low mobility characteristics can improve the recovery of ordinary heavy oil by polymer flooding. Therefore, it is proposed that an enhanced profile control and plugging effect due to the dual low mobility characteristics is an important EOR mechanism for ordinary heavy oil development by polymer flooding.

## 1 Introduction

Globally, heavy oil resources represent a significant portion of the world’s oil reserves, up to 25% according to some estimates.^1,2^ According to statistics, the proven reserves of heavy oil resources in the world are about 3000 × 10^8^ tons, mainly distributed in Canada, Venezuela, the United States, Russia, China, and Indonesia.^3^ Among them, China’s total heavy oil reserves exceed 200 × 10^8^ tons.^4,5^ With the continuous exploitation of oil resources, the recoverable reserves of conventional oil and gas are gradually decreasing. Through effective development, it can become a strategic alternative to conventional crude oil.^6^

For ordinary heavy oil (with viscosities of 100–10,000 cp), thermal recovery technology is generally used after primary oil recovery.^7−9^ Thermal recovery technology began in the 1930s, represented by applications in the United States and Canada, and four techniques have been successively developed—steam flooding, steam huff and puff, fired oil layer, and steam-assisted gravity drainage (SAGD)—which are mainly used in medium-shallow (<600 m) and thick-layer heavy oils.^10^ The mechanism of thermal recovery technology is mainly to reduce the viscosity of heavy oil to increase its fluidity. However, the application of thermal recovery technology has some crucial challenges due to its economic and environmental obstacles, especially in North America, Latin America, the Middle East, China, and so on.^11^ For example, SAGD is a thermal process that requires energy to convert water into steam, which is commercially expensive. In addition, when the oil formation is too thin (<10 m) or the reservoir is too deep (>1000 m) or there is bottom water, heat loss is severe, energy recovery efficiency is extremely low, and development costs increase significantly. This reduces the effectiveness of thermal recovery techniques to improve recovery rates, and there is an urgent need to change the way such fields are developed.^12^ For conventional heavy oil, which is not economically suitable for thermal recovery technology, chemical flooding is one of the most promising methods for enhanced oil recovery (EOR).^13^ Compared to other EOR methods, chemical flooding does not require expensive surface facilities. Therefore, heavy oil cold recovery technology has attracted increasing attention due to its advantages of low cost, low emission, energy saving, and environmental protection.^14^

In the process of heavy oil cold production, due to the problems of high viscosity, unfavorable mobility, and high viscosity ratio of oil to water, it is easy to produce an obvious fingering phenomenon, resulting in small effective swept volume of the displacement phase.^15,16^ A water-soluble polymer can effectively improve the mobility ratio, expand the swept volume of the water phase, and improve the efficiency of oil displacement.^17^ As early as the 1960s, Bleakley^18^ reported that polymer flooding for heavy oil reservoirs had been tested in the field. Elliot and Jose^19^ also reported the laboratory study of polymer flooding of heavy oil in the 1970s. With the gradual development of the technology, many oil fields have conducted field tests of polymer flooding of heavy oil.

In Oman, the Marmul field underwent a large-scale polymer flooding field test for 90 cp crude.^20,21^ The field began polymer flooding in 2010, and by 2013, the field’s water cut had decreased by 10%, and production had increased by approximately 25%. An oil field in China’s Bohai Bay^22^ has oil viscosities ranging from 30 to 450 cp. From 2003 to the present, polymer flooding has been carried out from a single well to a well pattern pilot test to an integrated oil field, gradually increasing the scale of testing. The total oil production improvement by the end of 2010 was more than 6.0 million bbl. In Canada, the largest existing heavy oil polymer flood at Pelican Lake has hundreds of injection wells in an oil with a mobile oil viscosity ranging from 800 to over 10,000 cp.^23,24^ Despite this high viscosity, a polymer flood pilot was conducted under secondary conditions and proved to be very successful, increasing the oil rate from 43 bopd to over 700 bopd while keeping the water cut below 60%. Polymer flooding is becoming recognized as an efficient and attractive process for increasing recovery in heavy oil reservoirs, following the success of pilots and even field-scale extensions in medium- to high-viscosity oil in several countries. As a result, a relatively extensive experimental study of polymer flooding of conventional heavy oil has been carried out both domestically and overseas.^25^

Buchgriber^26^ compared the microscopic displacement phenomena of a conventional polymer and associative polymer when displacing heavy oil with a viscosity of 210 cp, and the results showed that both polymers could inhibit the obvious fingering phenomenon produced during water flooding. Wassmuth and Xu^27,28^ conducted an experimental study on polymer flooding of heavy oil reservoirs with viscosities ranging from 300 to 1600 cp, and the results showed that polymer flooding can further improve the recovery of heavy oil based on water flooding. Wang and Dong^29^ demonstrated that there is an optimum viscosity range of the polymer solution during polymer flooding of heavy oil. Within this range, the recovery increases rapidly with the increasing polymer concentration, but when the concentration exceeds the optimum value, the increase in recovery slows down. Hou^30^ proposed that the water/oil mobility ratio was improved after polymer flooding, which resulted in the breaking of the “equilibrium” flow field formed during water flooding and the redistribution of the oil saturation regions. Lu^31^ studied the mechanism of EOR by polymer flooding in heterogeneous reservoirs. The results show that the main mechanism of EOR by polymer flooding is the expansion of the water phase sweep volume due to the retention of the polymer in porous media. Previous studies have shown that polymer flooding is feasible and has practical significance in the EOR of conventional heavy oil reservoirs. However, the understanding of its EOR mechanism is still dominated by polymer flooding of light oil. In conclusion, it is necessary to further investigate the oil displacement mechanism in the process of polymer flooding of heavy oil.

First, the injectable properties of polymers are experimentally studied to determine a reasonable concentration range for polymer solutions to be injected into experimental cores. In addition, the characteristics of the fluid seepage and oil displacement mechanism are analyzed in combination with the relative permeability curve test experiment and the oil displacement efficiency evaluation experiment. Furthermore, it is investigated that the effect of mobility control influences the oil displacement effect in the process of polymer flooding of ordinary heavy oil. Finally, based on the microscopic oil displacement experiment, we further discuss the mechanism in this process from a wide perspective.

## 2 Experimental Materials, Instruments, and Methods

### 2.1 Experimental Materials

The materials used in the experiment are as follows: Oil sample 1 is from the Daqing Oilfield, and the viscosity of the crude oil at 50 °C is 238.5 cp. Oil sample 2 is from the Daqing Oilfield, and the viscosity of the crude oil at 50 °C is 462.7 cp. The experimental water was formation water with a total salinity of 3619 mg/L; its mineral composition is shown in Table 1. The polymer used in the experiment is polyacrylamide with a molecular weight of 21 million, and its effective composition is 90%.

**Table 1:** Ion Composition Analysis^a^

	cation (mg/L)			anion (mg/L)				
ionic composition	Na^+^	Ca^2+^	Mg^2+^	HCO_3_^2–^	Cl^–^	SO_4_^2-^	CO_3_^2-^	total salinity (mg/L)
formation water	1152	166.5	117.5	1121.5	439.5	176	446	3619

a Experimental rock core: ① Rock core samples were obtained from the Daqing Oilfield. Eleven cores (L-1–L-11) were obtained by boring, which were then polished, cleaned, and dried. The length and diameter of each core were about 7.5 and 2.5 cm, respectively, and the permeability was about 1500 × 10^–3^ μm^2^ measured by gas. ② Six synthetic quartz sand epoxy resin-cemented homogenized cores were prepared (B-1–B-6). The dimensions of these cores were 20 cm × 5.5 cm × 5.5 cm, and the permeability was about 1500 × 10^–3^ μm^2^ measured by gas.

### 2.2 Experimental Instruments

The main experimental equipment was a high-temperature, high-pressure core flow tester. This system was equipped with a two-cylinder constant-speed constant-pressure pump, a piston chamber, pressure sensors, core holders, and a constant-temperature box. Auxiliary equipment used in this experiment includes a hand pump, Brookfield viscometer, vacuum pump, timer, mixer, and measuring tubes. The microscopic infiltration model consists of a glass etching model, a micropump, a microscope, a high-speed photography system, and an image analysis system.

### 2.3 Experimental Methods

#### 2.3.1 Polymer Injection Capacity Test

Cores L-1–L-5 are used for the polymer injection capacity test. The experimental scheme is shown in Table 2. The experimental procedure is as follows: the natural core was vacuumed to saturate formation water, and the pore volume and porosity of the core were calculated. Polymer solution preparation: polymer solution with a mass concentration of 5000 mg/L is prepared first which is allowed to stand at room temperature for 24 h and then diluted to the solution of the required mass concentration for the experiment, and after shearing pretreatment, it is placed in the constant-temperature box for 2 h. The viscosity value of polymer solution of each concentration is measured, and the relationship curve between the viscosity and concentration of polymer solution is drawn. The prepared polymer solution is injected into the core at a rate of 0.3 mL/min, and the change of the pressure in the core and the flow at the outlet is observed. After the pressure is stable, the stable pressure and the flow at this time are recorded. After the experiment, the resistance coefficient is calculated through eq 1.
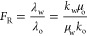
1where λ, μ, and *k* indicate the mobility ratio (dimensionless), viscosity (cp), and effective permeability (μm^2^), respectively. The subscripts “o” and “w” denote oil and aqueous phases, respectively.

**Table 2:** Experimental Scheme for the Polymer Injection Capacity Test

core number	length/(cm)	cross-sectional area/(cm^2^)	permeability/(10^–^^3^ μm^2^)	porosity/(%)	polymer solution concentration/(mg L^–1^)
L-1	7.51	4.91	1478	28.8	500
L-2	7.54	4.91	1503	28.65	1000
L-3	7.55	4.91	1516	28.72	1500
L-4	7.52	4.91	1498	28.78	2000
L-5	7.56	4.91	1486	29.07	2500

#### 2.3.2 Experiment on Measuring the Relative Permeability Curve by the Steady-State Method

Cores L-6–L-11 were used to determine the relative permeability curve. The specific experimental procedures are as follows: The natural core was vacuumed to saturate the formation water, and the pore volume and porosity of the core were calculated. An annular pressure of 5 MPa was applied to the core holder, and the sealing of the core holder was checked to see if it was normal. The core holder was placed at 50 °C for 3 days. Oil samples were pumped into the core at 0.1, 0.2, 0.5, and 1 mL/min until the water cut at the production end reached 0 (i.e., when the core was saturated with oil). After the pressure difference between the two ends of the core was stable, the corresponding saturation pressure at this time was recorded, and then, the oil phase permeability, original oil saturation, and irreducible water saturation under this condition were calculated. The formula for calculating the oil-phase permeability is shown in eq 2. The speed of the oil phase and water phase (polymer solution) was set according to the total flow rate of 0.2 mL/min. The oil phase and water phase were injected into the core at the same time according to an oil–water flow ratio of 19:1. When the two-phase oil–water flow in the core was stable, the pressure difference at the inlet and outlet of the core and the volume of the oil phase and water phase at the outlet of the core were recorded. The total flow rate was kept unchanged at 0.2 mL/min, and the flow ratio of oil and water phases was changed to 9:1, 6:1, 3:1, 1:1, 1:3, 1:6, 1:9, and 1:19, and the above-mentioned steps were repeated until the end of the experiment. According to Darcy’s law, the effective permeability and relative permeability of oil and water phases were calculated by the pressure difference between the two ends of the core measured in the experiment when the flow was stable.^32^ The effective viscosity of the polymer solution was calculated by measuring the relationship between the seepage rate and the shear rate of the polymer solution in the pore medium. The material balance method was used to measure the oil saturation and water saturation of the core under each oil–water flow ratio, draw the relative permeability curve, and calculate the mobility ratio. The above-mentioned steps were repeated to complete all experiments according to the experimental scheme shown in Table 3.
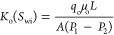
2where *K*_o_(*S*_wi_) denotes the effective permeability of the oil phase under the bound water condition (μm^2^); *q*_o_ represents the oil phase flow rate (mL/s); μ_o_ denotes the viscosity of the oil at the measured temperature (cp); *L* is the core length (cm); A represents the core cross-sectional area (cm^2^); and *P*_1_ – *P*_2_ represents the pressure difference between the inlet and outlet of the core (MPa).

**Table 3:** Experimental Scheme for Relative Permeability Curve Testing

core number	length/(cm)	cross-sectional area/(cm^2^)	permeability/(10^–^^3^ μm^2^)	porosity/(%)	viscosity of crude/(cp)	Polymer solution concentration/(mg·L^–1^)
L-6	7.54	4.91	1491	28.44	238.5	1000
L-7	7.58	4.91	1509	28.98	238.5	1500
L-8	7.57	4.91	1481	29.34	238.5	2000
L-9	7.54	4.91	1495	28.86	462.7	1000
L-10	7.51	4.91	1513	29.07	462.7	1500
L-11	7.55	4.91	1524	28.86	462.7	2000

#### 2.3.3 Oil Displacement Efficiency Evaluation Experiment

Cores B-1–B-6 were used for the oil displacement efficiency evaluation experiment. The experimental scheme is shown in Table 4. The specific experimental procedures are as follows:^33^ The artificial core was vacuumed to saturate the formation water, and the pore volume and porosity of the core were calculated. The oil sample was pumped into the core at a rate of 0.2 mL/min until the water cut at the production end reached 0 (i.e., when the core was saturated with oil). The original oil saturation of the rock was then calculated. The core holder was placed at 50 °C for 3 days. The formation water was pumped into the core at a rate of 0.3 mL/min until the water cut of the produced liquid at the core outlet reached 98%. Then, 0.4PV polymer solution was injected at the same rate. Finally, the formation water was pumped into the core at the rate of 0.3 mL/min until the water cut of the produced liquid at the core outlet reached 98%. The volume of oil and water in the produced fluid was recorded, and the water drive recovery, polymer drive recovery, and subsequent water drive recovery were calculated. The formula for calculating the oil recovery is shown as follows.
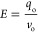
3where *E* represents the stage recovery degree (%), *q*_o_ represents the total amount of oil in produced liquid (mL), and *v*_o_ represents the total amount of saturated oil in the core (mL).

**Table 4:** Experimental Scheme of Oil Displacement Efficiency Evaluation

core number	length/(cm)	cross-sectional area/(cm^2^)	permeability/(10^–^^3^ μm^2^)	porosity/(%)	viscosity of crude/(cp)	Polymer solution concentration/(mg·L^–1^)
B-1	300	20.25	1506	31.01	238.5	1000
B-2	300	20.25	1507	30.89	238.5	1500
B-3	300	20.25	1504	31.28	238.5	2000
B-4	300	20.25	1511	30.73	462.7	1000
B-5	300	20.25	1498	29.91	462.7	1500
B-6	300	20.25	1500	30.82	462.7	2000

#### 2.3.4 Microscopic Oil Displacement Experiment

**Figure 1:**
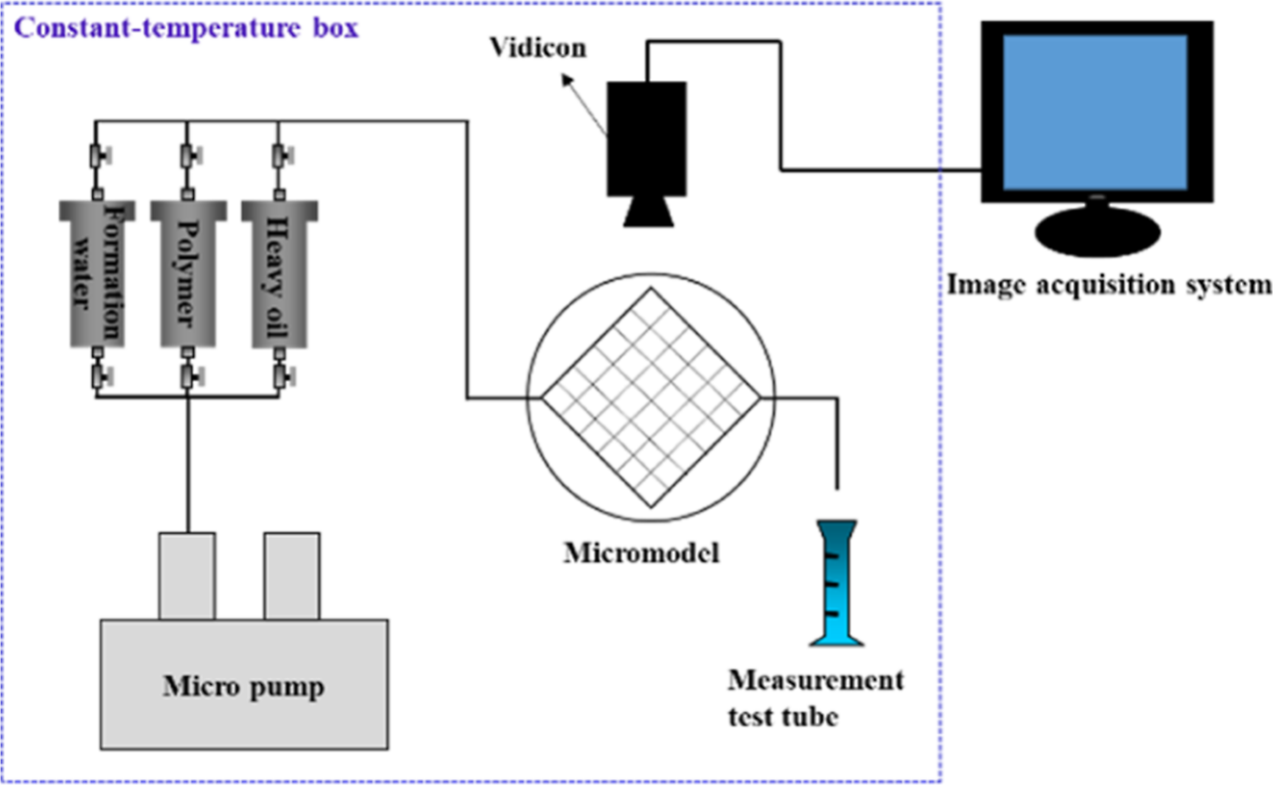
Experimental device diagram.

The specific experimental procedures are as follows: The apparatus was connected according to the apparatus diagram shown in Figure 1. The formation water was saturated after the microscopic model was vacuumized. The oil sample was pumped into the microscopic model at a rate of 0.03 mL/min until the water cut at the production end reached 0 (i.e., when the core was saturated with oil). The core holder was placed at 50 °C for 1 day. The formation water was pumped into the microscopic model at the rate of 0.03 mL/min until the water cut of the produced liquid at the microscopic model outlet reached 98%. Then, 0.4PV polymer solution was injected at the same rate. Finally, the formation water was pumped into the microscopic model at the rate of 0.03 mL/min until the water cut of the produced liquid at the microscopic model outlet reached 98%. A camera system was used to record the displacement process.

## 3 Results and Discussion

### 3.1 Polymer Injection Capacity Test Results

**Figure 2:**
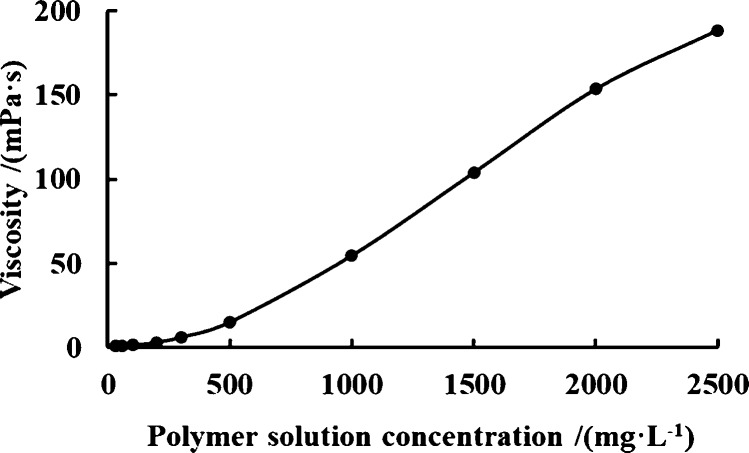
Relationship between the concentration and viscosity of polymer solution.

The relationship between the polymer solution concentration and viscosity at 50 °C is shown in Figure 2. The viscosity of the polymer solution takes 300 mg/L as the boundary and shows two growth trends. When the polymer concentration is less than 300 mg/L, the viscosity of the polymer solution grows slowly and approximately linearly. When the polymer concentration reaches 300 mg/L, the viscosity of the polymer solution increases rapidly. That is because polymers are long-chain, high-molecular-weight compounds, and their viscosity-increasing performance is mainly achieved by molecular chain association, winding, and folding. In low-concentration polymers, this kind of intermolecular interaction is weak, and the viscosity change range is small. However, when the concentration of the polymer solution reaches a critical value, the probability of molecular chain collision and winding increases, and the viscosity of the polymer solution rises sharply when the polymer concentration exceeds this critical value.

In addition, previous studies have shown that a higher polymer concentration can achieve higher recovery. In general, there is an optimal range of polymer concentrations, in which the recovery rate increases rapidly with the increase of the polymer concentration, and then, the recovery rate increases slowly. Therefore, the concentration of polymer solution should be at least 300 mg/L. However, for the effective displacement of ordinary heavy oil, polymer optimization should be carried out on the premise of ensuring the effective injection of the polymer. At the same time, this paper mainly studies the mobility control ability of polymers, so the upper limit of the polymer concentration should be determined by the results of the injection capacity test.

The polymer injection capacity test results are shown in Table 5. The polymer solution of 500–2000 mg/L has a good injectivity, but the polymer solution of 2500 mg/L has a poor injectivity due to its high viscosity. At the same time, the higher the concentration of polymer solution, the higher the resistance coefficient, and the better the mobility control effect, and it can effectively expand the sweep volume of the water phase. Therefore, polymer solutions with concentrations of 1000, 1500, and 2000 mg/L were selected for the relative permeability experiment and the oil displacement efficiency evaluation experiment.

**Table 5:** Polymer Solution Injection Capacity Test Results

core number	stable pressure difference/(MPa)	polymer solution concentration/(mg L^–1^)	resistance factor	whether it passes or not	whether it will clog the core
L-1	0.22	500	3.24	Yes	no
L-2	0.31	1000	8.42	Yes	no
L-3	0.42	1500	14.13	Yes	no
L-4	0.63	2000	17.86	Yes	no
L-5	2.08	2500	25.09	No	yes

### 3.2 Analysis of Mobility Control Ability

**Figure 3:**
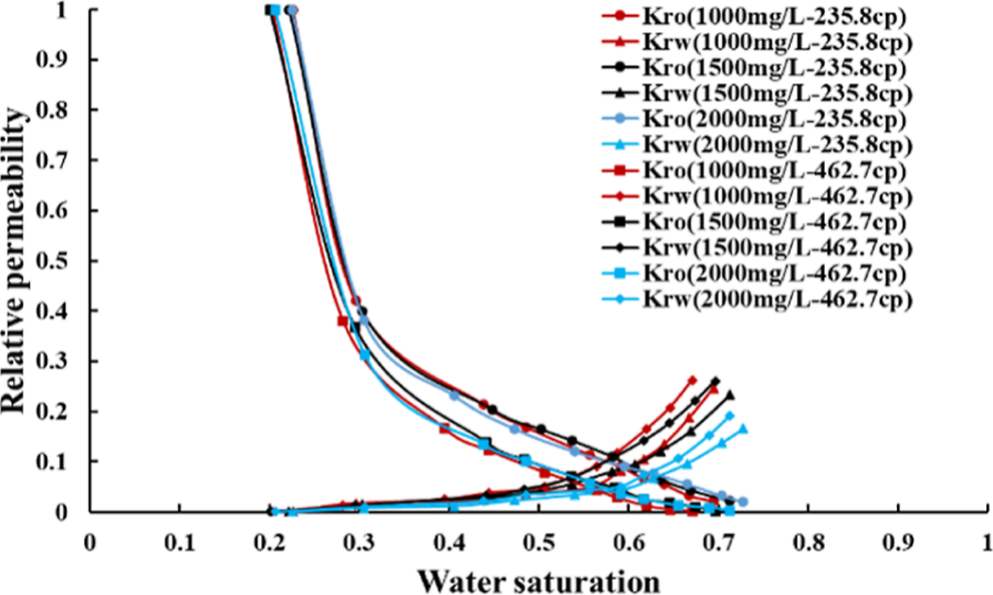
Polymer–ordinary heavy oil relative permeability curves [take the first curve in the legend as an example: 1000 mg/L-235.8 cp represents the polymer (concentration of 1000 mg/L)—ordinary heavy oil (viscosity of 235.8 cp) relative permeability curve].

The two-phase relative permeability curve of the polymer and ordinary heavy oil is shown in Figure 3. Under the condition of the same crude oil viscosity, as the viscosity of the polymer solution increases, the area of the oil–water two-phase permeation zone becomes larger, the residual oil saturation decreases, the relative permeability of the water phase decreases, and the relative permeability of the oil phase hardly changes. Therefore, the higher the polymer concentration, the higher the viscosity, the better the effect of improving the oil–water mobility ratio, the better the mobility control effect of the water phase during polymer flooding, and the higher the oil displacement efficiency. When the concentration of the polymer solution is the same, as the crude oil viscosity increases, the area of the oil–water two-phase permeation zone becomes smaller, the residual oil saturation increases, and the relative permeability of the oil–water two-phase zone decreases. This is because the viscosity ratio of oil–water increases, the seepage resistance increases, the effective sweep volume of the displacement phase decreases, the relative permeability of the oil–water two-phase zone decreases, and the mobility ratio of the displacement phase to the displaced phase increases so that the oil displacement effect is weakened.

**Figure 4:**
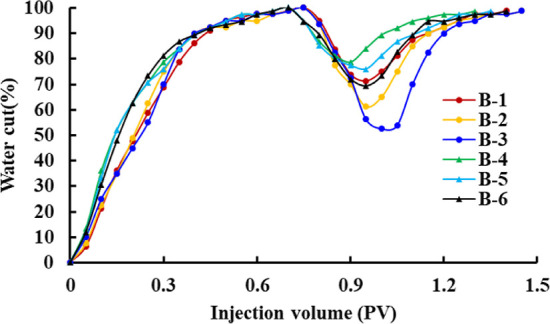
Water cut change curves of schemes B-1–B-6.

**Figure 5:**
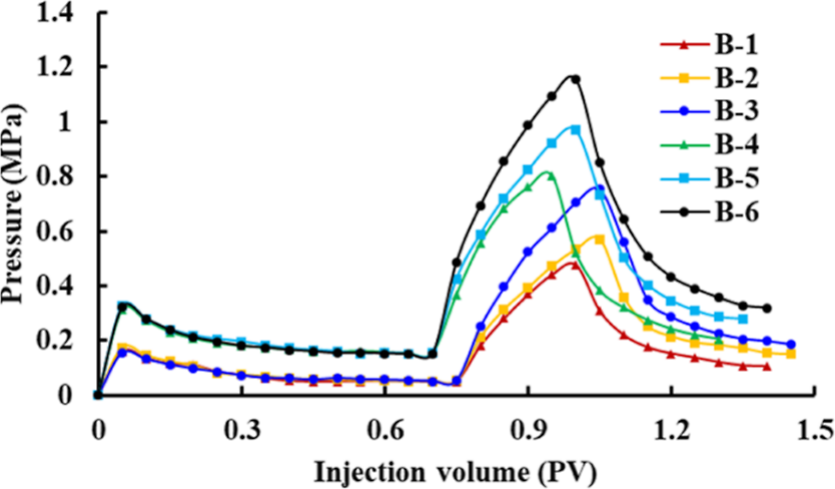
Pressure change curves of schemes B-1–B-6.

**Figure 6:**
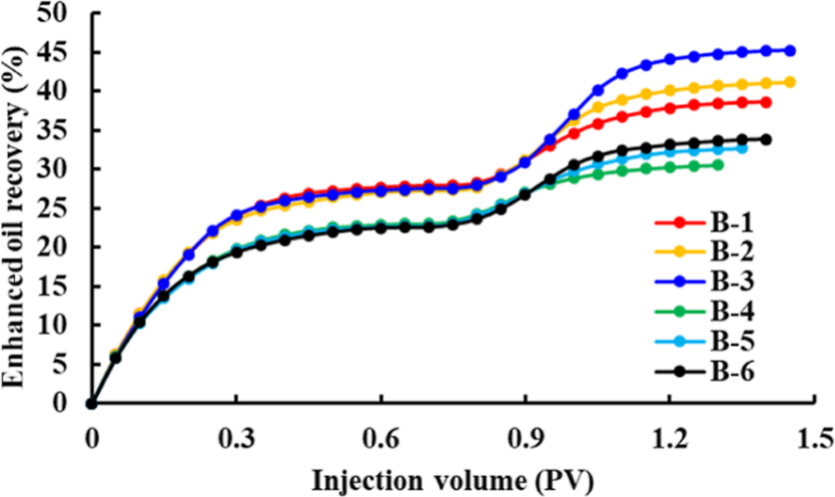
Recovery change curves of schemes B-1–B-6.

The change curves of recovery, water cut, and pressure for schemes B-1–B-6 are shown in Figures 4–6. In the water flooding stage, due to the high viscosity of heavy oil and the oil–water two-phase mobility ratio being large, an obvious fingering phenomenon will occur in the process of water injection development. The water cut will rapidly increase to about 95%, and the pressure will slowly decrease after the rapid increase and then become stable. At the same time, the higher the viscosity of crude oil, the higher the initial pressure gradient, the faster the water cut and pressure rise, and the lower the displacement efficiency. In the polymer flooding stage, when polymer solutions with concentrations of 1000, 1500, and 2000 mg/L displace ordinary heavy oil with a viscosity of 235.8 cp, the water cut decreases to 71.25, 61.25, and 52.5%, respectively. The pressure increases to 0.478, 0.569, and 0.753 MPa, respectively. When the polymer solutions with concentrations of 1000, 1500, and 2000 mg/L displace the ordinary heavy oil with a viscosity of 462.7 cp, the water cut decreases to 78.7, 76, and 69.3%, respectively, and the pressure increases to 0.804, 0.968, and 1.153 MPa, respectively. Thus, it can be seen that when the viscosity of the crude oil is the same, the higher the viscosity of the polymer solution, the larger the water cut reduction range, the faster the rate of rise of the pressure curve, and the higher the oil displacement efficiency. When the viscosity of the polymer solution is the same, the higher the viscosity of the crude oil, the smaller the water cut reduction range, the slower the rate of rise of the pressure curve, and the lower the oil displacement efficiency.

The results of the oil displacement efficiency evaluation experiment and the mobility ratio calculation are shown in Table 6. When the polymer solution with a concentration of 2000 mg/L displaces the crude oil with a viscosity of 238.5 cp, the water–oil two-phase mobility ratio is only 0.409. At this time, the oil displacement effect is the best, and the EOR after polymer flooding can reach 14.69%. When the polymer solution with a concentration of 1000 mg/L displaces the crude oil with a viscosity of 462.7 cp, the water–oil two-phase mobility ratio reaches 2.248, and the oil displacement effect is relatively poor, but the oil recovery can still be improved by 6.44% after polymer flooding. In the process of polymer flooding, the mobility ratio of water–oil two-phase is small, far less than that of water–oil two-phase in the process of water flooding; this extremely low mobility ratio has a significant impact on the oil displacement effect. Therefore, this paper based on the characteristics of high viscosity and low mobility of polymer solution and heavy oil proposes that the existence of dual low mobility characteristics in the process of polymer flooding of ordinary heavy oil is an important factor to effectively improve oil recovery.

**Table 6:** Results of the Oil Displacement Efficiency Evaluation Experiment and Mobility Ratio Calculation

core number	initial oil saturation/(%)	recovery degree in the water flooding stage/(%)	recovery degree in the polymer flooding stage/(%)	recovery degree in the subsequent water flooding stage/(%)	mobility ratio
B-1	74.08	27.92	8.81	1.91	1.146
B-2	74.16	27.31	11.67	2.2	0.604
B-3	74.53	27.53	14.69	3.02	0.409
B-4	72.06	23	6.44	1.1	2.248
B-5	72.82	22.75	7.94	2.05	1.185
B-6	72.66	22.55	9.2	2.12	0.803

### 3.3 Micro-Seepage Characteristics

**Figure 7:**
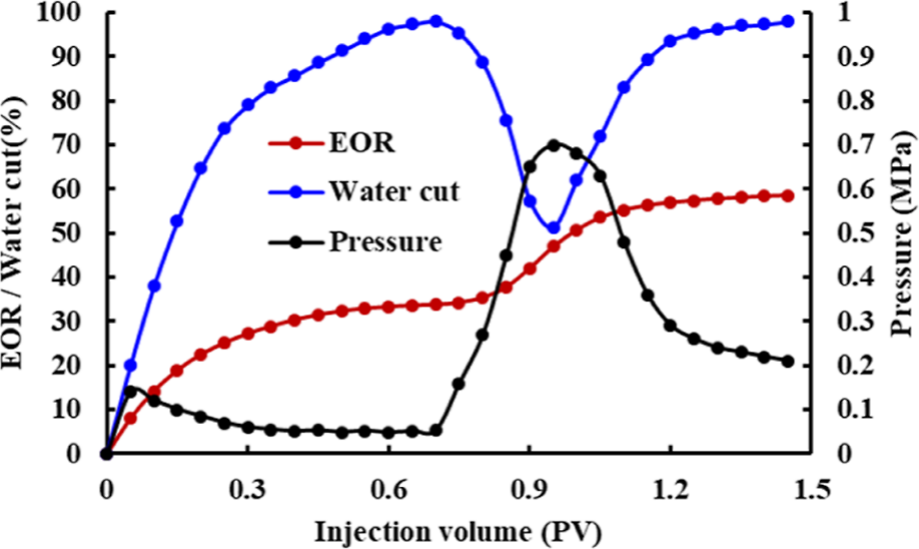
Curves of recovery, water cut, and pressure in the micro-displacement experiment.

The micro-flooding experiment of polymer flooding of ordinary heavy oil was carried out. First, water flooding is carried out until the water cut in the produced liquid reaches 98%, and then, 0.4PV polymer flooding is carried out. The viscosity of the oil used in the experiment is 238.5 cp, and the concentration of the polymer solution is 2000 mg/L. The variation curves of recovery, water cut, and pressure in the microscopic oil displacement experiment are shown in Figure 7. It can be seen from the curves that the curve rules of the polymer micro-displacement experiment and macro-oil displacement experiment are similar. In the water flooding stage, the water cut rapidly increases to 98%, the pressure rapidly increases and then slowly decreases and becomes stable, and the recovery efficiency gradually increases to 33.77%. After polymer injection, the water cut rapidly decreased to 51.33%, the pressure rapidly increased to 0.7 MPa, and the recovery efficiency gradually increased to 55.23%. In the subsequent water flooding stage, the water cut rapidly increased to 98%, the pressure rapidly decreased and then stabilized, and the recovery rate gradually increased to 58.53%.

**Figure 8:**
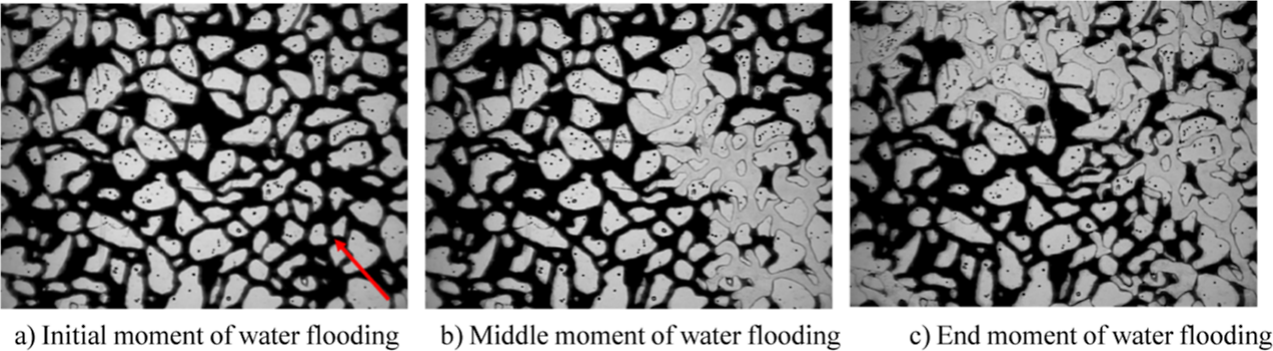
Microscopic percolation characteristics of water flooding of ordinary heavy oil.

**Figure 9:**
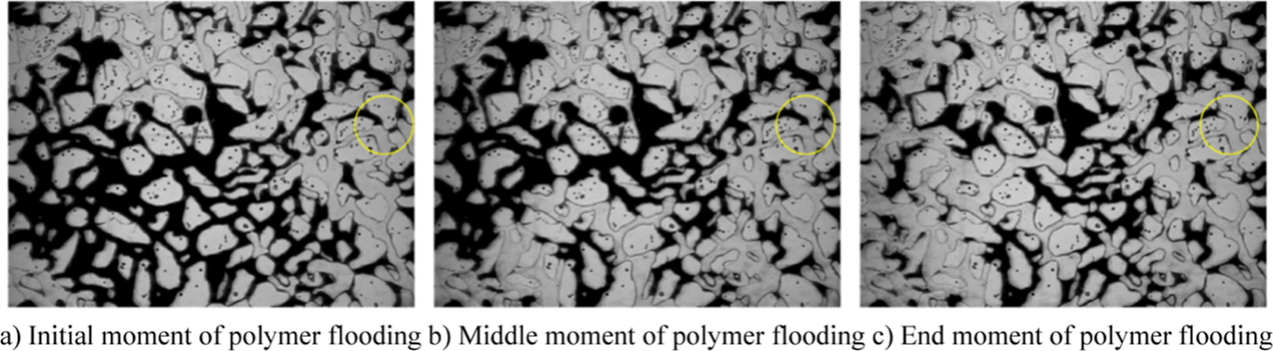
Microscopic percolation characteristics of polymer flooding of ordinary heavy oil.

The experimental process is shown in Figures 8 and 9, where the red mark in Figure 8a is the injection direction of the displacement phase fluid. As can be seen in Figure 8, in the process of water flooding, the fingering phenomenon is obvious, the sweep volume is small, and the dominant water flow channel is rapidly formed. This is due to the high oil–water viscosity ratio and high seepage resistance in ordinary heavy oil–water flooding. In addition, a large amount of injected water enters an invalid circulation state, where it cannot reach areas outside the dominant water flow channel. Therefore, the overall oil displacement efficiency of water-driven ordinary heavy oil is low.

Figure 9 shows that polymer flooding of ordinary heavy oil effectively alleviates the fingering phenomenon in the process of water flooding, and the sweep range of the water phase after polymer injection increases, the profile control effects are significant, and the remaining oil near the injection end is obviously recovered. This is because the viscosity of the water phase increases, and the oil–water mobility ratio decreases after polymer injection. At the same time, the ability of the water phase to carry liquid is enhanced by the viscoelasticity of the polymer, and the remaining oil in the large pore channel in the sweep area of the water phase is driven out. As can be seen from the yellow marks, after the remaining oil in the larger pore is driven out by the polymer solution, the further injected polymer solution will preferentially enter the larger pore and remain in the larger throat under the effect of mechanical trapping, resulting in additional seepage resistance. When the injection rate is kept constant, the suction fluid pressure difference and absorption amount of the small pore around are significantly increased, and the remaining oil in the small pore is driven out, thus improving the oil displacement efficiency. Therefore, under the dual low mobility characteristics, the profile control and plugging effect of the polymer have the synergistic EOR effect of increasing the sweep volume of larger channels and the utilization degree of smaller channels.

## 4 Conclusions

(1) Based on the high viscosity and low mobility characteristics of the polymer and ordinary heavy oil, it is proposed that there are dual low mobility characteristics in polymer flooding of ordinary heavy oil, which can effectively improve the mobility control effect of the displacement phase.
(2) In the process of polymer flooding of ordinary heavy oil, the polymer will preferentially enter the large pore channels, expand the sweep volume of the large channels, and plug the large pore channel by mechanical trapping, the significant profile control effect. Meanwhile, the fluid suction pressure difference and fluid absorption amount of the small pore around are significantly increased, and the remaining oil in the small pore is driven out.
(3) Under the influence of dual low mobility characteristics, the profile control and plugging effect of the polymer is enhanced. Therefore, it is proposed that the dual low mobility characteristic-enhanced profile control and plugging effect is one of the EOR mechanisms of polymer flooding of ordinary heavy oil.
